# Pelvic intraoperative neuromonitoring during robotic-assisted low anterior resection for rectal cancer

**DOI:** 10.1007/s11701-015-0556-6

**Published:** 2015-12-24

**Authors:** Marian Grade, Alexander W. Beham, P. Schüler, Werner Kneist, B. Michael Ghadimi

**Affiliations:** Department of General, Visceral and Pediatric Surgery, University Medical Center, Robert-Koch-Str. 40, 37075 Goettingen, Germany; Department of General, Visceral and Transplant Surgery, University Medical Center, Langenbeckstraße 1, 55131 Mainz, Germany

**Keywords:** Rectal cancer, Low anterior rectal resection, Robotic surgery, Nerve-sparing total mesorectal excision, Intraoperative neuromonitoring, Autonomic pelvic nerves

## Abstract

While the oncological outcome of patients with rectal cancer has been considerably improved within the last decades, anorectal, urinary and sexual functions remained impaired at high levels, regardless of whether radical surgery was performed open or laparoscopically. Consequently, intraoperative monitoring of the autonomic pelvic nerves with simultaneous electromyography of the internal anal sphincter and manometry of the urinary bladder has been introduced to advance nerve-sparing surgery and to improve functional outcome. Initial results suggested that pelvic neuromonitoring may result in better functional outcomes. Very recently, it has also been demonstrated that minimally invasive neuromonitoring is technically feasible. Because, to the best of our knowledge, pelvic neuromonitoring has not been performed during robotic surgery, we report the first case of robotic-assisted low anterior rectal resection combined with intraoperative monitoring of the autonomic pelvic nerves.

## Introduction

The surgical concept of total mesorectal excision (TME) and the implementation of multimodal preoperative treatment strategies have considerably improved the oncological outcome of patients with rectal cancer [1]. However, it became evident that, despite prolonged survival rates, anorectal, urinary and sexual functions remained impaired at high levels [2–4], regardless of whether TME was performed as open or laparoscopic surgery [5]. Whether robotic-assisted surgery leads to improved functional outcome remains unclear, as the results of the prospective randomized ROLARR trial, which compares robotic-assisted versus standard laparoscopic anterior rectal resection, have not yet been published [6].

Monitoring of the autonomic pelvic nerves with simultaneous electromyography of the internal anal sphincter and manometry of the urinary bladder has been introduced to advance nerve-sparing surgery and to improve functional outcome [7–9]. Initial results suggest that intraoperative neuromonitoring (IONM) may result in lower rates of urinary and anorectal dysfunction [7], and may predict male erectile dysfunction [8]. Very recently, it has also been demonstrated that minimally invasive IONM is technically feasible [10]. Because, to the best of our knowledge, monitoring of the autonomic pelvic nerves has not been performed during robotic surgery, we report the first case of robotic-assisted low anterior rectal resection combined with IONM.

## Case report

Our patient is a 58-year old man who was diagnosed with an adenocarcinoma of the rectum, located 8 cm above the anal verge. By means of magnetic resonance tomography and endorectal ultrasound, the tumor was classified as T1 without evidence for lymph node involvement. Computed tomography excluded distant metastases. Otherwise, he was healthy with a body mass index of 24.6. After discussion in the institution’s interdisciplinary tumor board, he was referred to surgery, which we performed using the da Vinci^®^ Si Surgical System.

## Operative technique and IONM

**Fig. 1:**
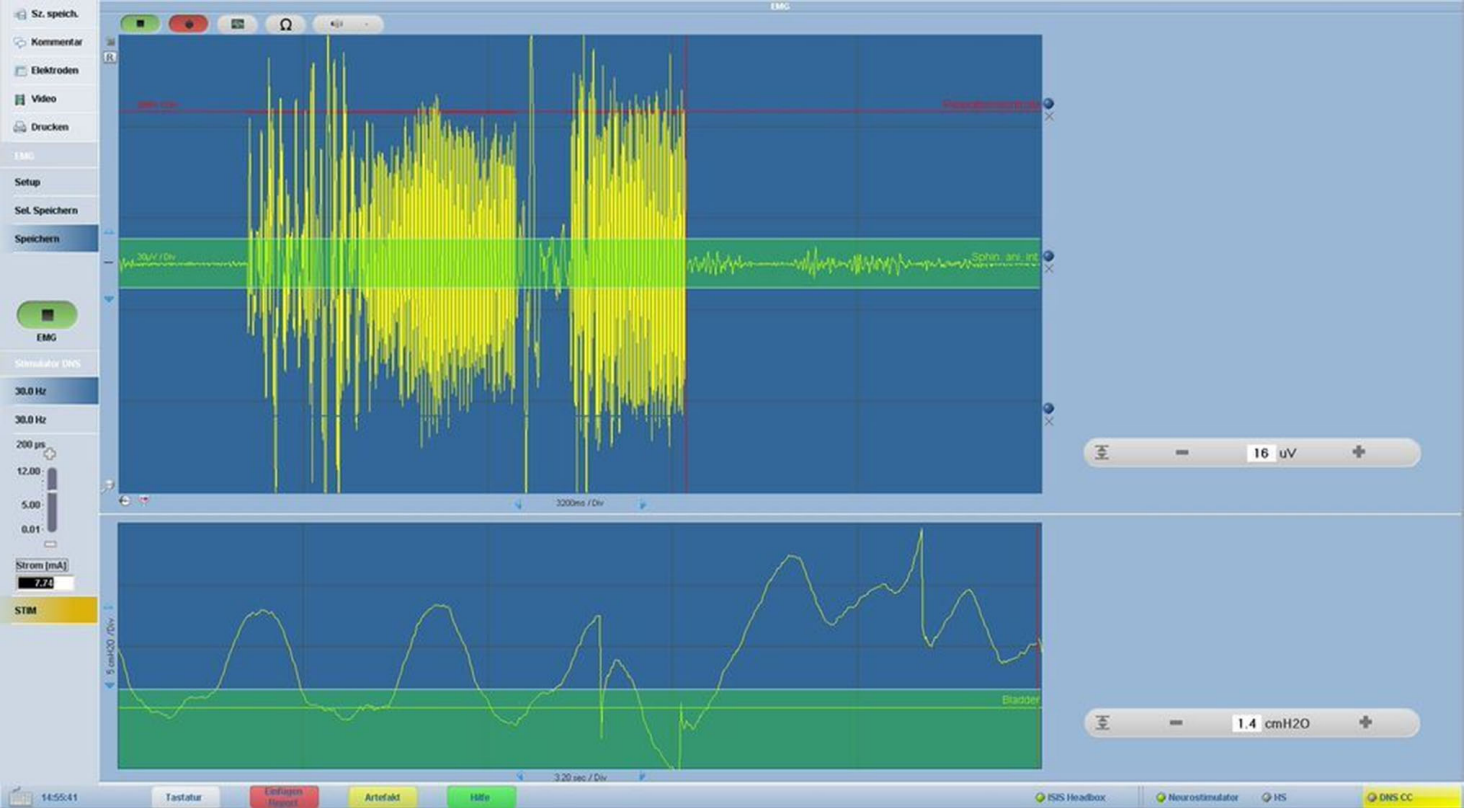
Simultaneous electromyography of the internal anal sphincter (*upper panel*) and manometry of the urinary bladder (*lower panel*)

Preoperatively, the patient underwent a bowel preparation, and the operation was performed under inhalation anesthesia without a thoracic epidural catheter. The patient was placed in a modified lithotomy and Trendelenburg position, slightly tilted to the right side. For electromyography of the internal anal sphincter, bipolar needle electrodes (inomed Medizintechnik GmbH, Emmendingen, Germany) were placed into both the internal and external sphincters, and a reference electrode was placed into the left gluteal muscle, as reported in detail by Kneist and colleagues [8–10]. For manometry of the urine bladder, a transurethral catheter was connected, together with the electromyography cable, to a monitoring device (inomed Medizintechnik GmbH), allowing to control both IONM signals simultaneously on one screen (Fig. 1). As previously recommended, the following parameters were used for nerve stimulation: currents of 6 mA, frequency of 30 Hz, and monophasic rectangular pulses with pulse duration of 200 μs [8–10].

A 12-mm optic trocar was inserted slightly right craniolaterally of the umbilicus by means of a minilaparotomy. After the appearance of adhesions had been excluded, two 8-mm da Vinci^®^ trocars were placed on the left side, followed by an 8-mm da Vinci^®^ and a 12-mm assistant trocar on the right side. After establishment of a capnoperitoneum, the da Vinci^®^ patient cart was docked.

The sigmoid and descending colon were mobilized in a medial to lateral approach, and the left ureter was identified. Next, the inferior mesenteric vessels were ligated, followed by pelvic dissection of the rectum with TME. The rectum was mobilized down to a level just above the levator ani muscle. During and after dissection of the mesorectum, the pelvic autonomic nerves were monitored bilaterally using a bipolar microfork probe (inomed Medizintechnik GmbH). As shown in Fig. 1, simultaneous electromyography of the internal anal sphincter (upper panel) and manometry of the urinary bladder (lower panel) were successfully conducted. The rectum was diverted with an endoscopic linear stapler, and after extracorporeal division of the descending colon, a stapled anastomosis was created. The operation was finished by creation of a diverting loop-ileostomy.

## Discussion

The anatomy of the pelvis is highly complex, particularly with respect to the autonomic nervous system and its interindividual variations [11–15]. Accordingly, a profound anatomical knowledge is required for surgical procedures in the narrow pelvis. It is anticipated that pelvic nerve injury may occur at different sites, with varying functional consequences [13–15]: First, during ligation of the mesenteric inferior artery at its origin from the aorta (superior hypogastric plexus); second, during posterior rectal dissection when separating the parietal (presacral) and the visceral (perirectal) pelvic fascia at the level of the sacral promontory (hypogastric nerves); third, during anterolateral rectal dissection close to the lateral ligaments (inferior hypogastric plexus); fourth, during anterior rectal dissection at the lateral edge of Denonvilliers’ fascia (urogenital neurovascular bundles).

In this respect, nerve-sparing TME is complicated by the fact that neural structures, particularly the inferior hypogastric plexus and the pelvic splanchnic nerves with their complex network of fibers, are difficult to identify macroscopically. Consequently, there was a great hope that anorectal, urinary and sexual functions would be improved after laparoscopic TME, due to enhanced vision in the small pelvis compared to open surgery. However, the COLOR II randomized trial, which compared laparoscopic and open surgery for rectal cancer, reported very recently that there were no differences between the two groups with respect to genitourinary dysfunction, while micturition symptoms were less affected than sexual function [5]. Because the results of the ROLARR trial have not yet been published [6], it remains unclear whether robotic-assisted surgery may improve the functional outcome.

Therefore, IONM may be of substantial value, because it provides the surgeon with a direct feedback whether the plane of dissection is close to the pelvic autonomic nerves. Initial monocentric reports were promising and suggested potential benefits for the patient [7–9]. While Kneist and colleagues demonstrated that pelvic neuromonitoring can be performed during laparoscopic TME [10], we now report the first case of robotic-assisted low anterior rectal resection combined with IONM of the pelvic nerves. Up to now, we have performed three cases without any relevant problems.

## Conclusion

Intraoperative monitoring of the autonomic pelvic nerves is technically feasible during robotic-assisted low anterior rectal resection. Its definitive value to preserve urinary, anorectal and sexual function remains to be elucidated.
